# Model cerebellar granule cells can faithfully transmit modulated firing rate signals

**DOI:** 10.3389/fncel.2014.00304

**Published:** 2014-10-13

**Authors:** Christian Rössert, Sergio Solinas, Egidio D'Angelo, Paul Dean, John Porrill

**Affiliations:** ^1^Department of Psychology, University of SheffieldSheffield, UK; ^2^Brain Connectivity Center, Istituto Neurologico Istituto di Ricovero e Cura a Carattere Scientifico C. MondinoPavia, Italy; ^3^Laboratory of Neurophysiology, Department of Brain and Behavioural Sciences, University of PaviaPavia, Italy

**Keywords:** transfer function, Wiener filter, signal reconstruction, variance-accounted-for, mossy fiber, modulation

## Abstract

A crucial assumption of many high-level system models of the cerebellum is that information in the granular layer is encoded in a linear manner. However, granule cells are known for their non-linear and resonant synaptic and intrinsic properties that could potentially impede linear signal transmission. In this modeling study we analyse how electrophysiological granule cell properties and spike sampling influence information coded by firing rate modulation, assuming no signal-related, i.e., uncorrelated inhibitory feedback (open-loop mode). A detailed one-compartment granule cell model was excited in simulation by either direct current or mossy-fiber synaptic inputs. Vestibular signals were represented as tonic inputs to the flocculus modulated at frequencies up to 20 Hz (approximate upper frequency limit of vestibular-ocular reflex, VOR). Model outputs were assessed using estimates of both the transfer function, and the fidelity of input-signal reconstruction measured as variance-accounted-for. The detailed granule cell model with realistic mossy-fiber synaptic inputs could transmit information faithfully and linearly in the frequency range of the vestibular-ocular reflex. This was achieved most simply if the model neurons had a firing rate at least twice the highest required frequency of modulation, but lower rates were also adequate provided a population of neurons was utilized, especially in combination with push-pull coding. The exact number of neurons required for faithful transmission depended on the precise values of firing rate and noise. The model neurons were also able to combine excitatory and inhibitory signals linearly, and could be replaced by a simpler (modified) integrate-and-fire neuron in the case of high tonic firing rates. These findings suggest that granule cells can in principle code modulated firing-rate inputs in a linear manner, and are thus consistent with the high-level adaptive-filter model of the cerebellar microcircuit.

## Introduction

Understanding the function of the cerebellar microcircuit means relating the properties of its neural constituents to its overall computational capacity. Models are likely to prove useful for this purpose, but at present they often take one of two forms with little interaction between them. Detailed models of spiking neurons and networks are powerful tools for making contact with experiment, but the focus on low-level features such as channel properties can preclude computational analysis. Conversely, high-level lumped system models that do allow computational analysis typically lack the detail required to make meaningful contact with biology. Multi-scale modeling has been suggested as a natural approach to overcome these kinds of problems (e.g., Sejnowski et al., 1988; Noble, 2002; Hunter and Nielsen, 2005).

The first challenge in reconciling these two levels of modeling is to justify the simplification made from biological spike-coded signals to linear continuous signals. A natural starting point for this analysis in the cerebellum is the granular layer network. Here, the granule cells process the very extensive mossy-fiber input to the cerebellum, and are the most numerous type of neuron not only in the cerebellum itself, but also in the entire mammalian brain (e.g., Herculano-Houzel, 2010). They function as part of a recurrent network in the granular layer, which involves inhibitory feedback from Golgi cells, and our long-term goal is to characterize how this network as a whole can transform mossy fiber inputs.

Granule cells are known for their sub-threshold voltage-dependent potassium channels that promote resonance during sinusoidal and burst stimulation (D'Angelo et al., 2001; Gandolfi et al., 2013). Furthermore, mossy fiber to granule cell synaptic transmission is based on non-linear transformations determined by several pre- and postsynaptic mechanisms (Arleo et al., 2010). While it has been suggested that these intrinsic and synaptic mechanisms can improve spike timing of theta-frequency bursts (Gandolfi et al., 2013) their involvement in other coding schemes has not been examined yet.

Since the mossy-fiber input to the cerebellum arises from many different sources, it seems likely that the granular layer is able to deal with a wide variety of neural coding schemes. Initial recordings from granule cells *in vivo* support this view (Arenz et al., 2009). Here we focus on one particular coding scheme (Arenz et al., 2009; Galliano et al., 2010), namely modulated firing-rate (MFR) coding, where the dynamics of sensory signals are encoded as temporal modulation of a tonic firing-rate. The mossy-fiber vestibular input to the flocculus uses MFR (Arenz et al., 2008), and it is known that the flocculus is essential for calibrating the vestibulo-ocular reflex (VOR). The VOR has been extensively studied experimentally (for review see Boyden et al., 2004) and theoretically, both in the linear-systems framework (Robinson, 1981) and from the perspective of the adaptive-filter model of the cerebellum (Porrill and Dean, 2007; Dean et al., 2010).

Another interesting feature of granule cells is that these only possess a mean of 4 dendrites and that despite high activity of mossy fibers carrying MFR coded signals (e.g., 40 spikes/s flocculus, Lisberger and Fuchs, 1978) the activity of granule cells themselves is often reported to be irregular and low (Hensbroek et al., 2006; Barmack and Yakhnitsa, 2008). The effect of this “down sampling” on signal transmission fidelity however is currently unknown and might lead to imprecise signal transmission. To this end we also analyzed how “push-pull” coding, where two populations of the same cell type encode the positive and the negative amplitude respectively, can improve fidelity in the case of low firing rates.

To answer the question whether granule cells can faithfully and linearly represent vestibular-like input signals this study analyses information transmission by detailed granule cell models in open-loop mode, that is without input-related, i.e., uncorrelated, inhibitory feedback. This strategy was chosen following the assumption that information lost in open-loop mode cannot be regained whatever the properties of closed-loop mode.

Furthermore, this study serves as an initial step toward understanding the dynamical complexities of granular-layer processing: analysing signal transmission properties of granule cells in this study will help to separate intrinsic- and network contribution in future research.

In modeling the coding by floccular GrCs of vestibular inputs we can concentrate on the transmission of MFR signals as used in motor systems, and can ask whether the signal transmission properties of the model are consistent with data for real motor systems.

The main model analyzed here is that of D'Angelo et al. (2001), with mossy-fiber synaptic inputs as modeled by Nieus et al. (2006) and the minor modifications described by Solinas et al. (2010). This model has a single compartment and 10 active ion channels, and quantitatively reproduces a wide range of *in vitro* phenomena, including ionic current measurements, IPSC and EPSC kinetics, shape of action potential, and the timing and frequency of action potentials in response to current injection and synaptic stimulation (Solinas et al., 2010).

It appears that for natural head rotations in primate and human most of the power is below 20 Hz (Grossman et al., 1988; Pozzo et al., 1990; Demer and Viirre, 1996; Carriott et al., 2013). Appropriately, VOR performance in humans and primates is good up until (at least) 20 Hz (Tabak et al., 1997; Huterer and Cullen, 2002; Ramachandran and Lisberger, 2005). We therefore focussed on modeling GrC responses to input modulations up to 20 Hz.

## Materials and methods

### Single cell and synaptic models

The granule cell (GrC) model and corresponding AMPA, NMDA, and GABA synapse models used in all simulations are from Solinas et al. (2010). These models are based on the previous models of D'Angelo et al. (2001) (for GrC), Nieus et al. (2006) (for AMPA, NMDA synapses) and Mapelli et al. (2009) (for GABA_A_ synapse) with parameters appropriately adjusted for an operating temperature of 37°C (corresponding to *in vivo* rather than *in vitro* conditions). The detailed excitatory synaptic models explicitly simulate presynaptic depression using a three state scheme, presynaptic facilitation and postsynaptic depression due to receptor desensitization. The inhibitory synaptic models include fast direct activation of α_1_ and slow spillover activation of α_6_ GABA_A_ receptors. The granule cell and synaptic models have previously been made available on the Open Source Brain repository: http://github.com/OpenSourceBrain/GranCellSolinasEtAl10. Furthermore, the models and all scripts used to analyse the models were made available on the github repository https://github.com/croessert/AnalyseGranCellRoessertEtAl14. A snapshot of the scripts and models can also be found on modeldb: http://senselab.med.yale.edu/modeldb/ShowModel.asp?model=156733.

The GrC model has a single compartment to reflect the granule cell's compact electrotonic structure, with nine active conductances (3 sodium, 5 potassium, 1 calcium) and a non-specific leakage current. Conductances and calcium dynamics are modeled using standard methods (e.g., Yamada et al., 1998) and parameters based on experimental measurements given in Table 1 of D'Angelo et al. (2001). The model had a capacitance of *C* = 3 pF and the resistance, measured at rest by a hyperpolarizing current of −1pA, was *R* = 1049 MΩ giving a time constant of τ = 3.15 ms. The spike detection threshold was set to −20 mV in all simulations.

To help characterize the contribution of the complex synaptic and conductance properties of the detailed model to its information-processing capacities, its responses were compared with those of two simpler artificial neurons (e.g., Gabbiani and Koch, 1998). The first was a standard integrate-and-fire (IF) neuron, the second was a modified IF neuron including a resonant current *I*_*B*_ modeled as an abstract spike-dependent leak conductance (Benda and Herz, 2003) and a spike delay. The equation for the resonant neuron (rIF) was:

(1) $$\begin{array}{l} \;C\frac{dV}{dt}=-\frac{1}{R}(V-E_{R})-I_{B}+I_{E}\\ \text{with }I_{B}=g_{b}\cdot b(V-E_{R});\;\tau_{b}\frac{db}{dt}=\delta (t-t_{i})-b \end{array}$$

Output spikes were additionally delayed by a time Δ_*S*_ with respect to the input signal. This was done without additional effect on the membrane function (1) to reflect the pure phase delay in the estimated transfer function of the detailed model (see Results).

The equation for the standard IF neuron is obtained by setting *I*_*B*_ = 0 and Δ_*S*_ = 0. The stimulation current is *I*_*E*_, the term δ(*t* − *t*_*i*_) denotes the Dirac delta-function where *t*_*i*_ is the time of the latest spike. In this model the following parameters were obtained directly from the GrC model: *C* = 3 pF, resting and reset *E*_*R*_ = −71.5 mV (set to GrC resting potential), threshold potential *V*_th_ = −41.8 mV (set to give the same rheobase current-step threshold of 5.68 pA as the GrC model). The remaining parameters were estimated by fitting to the transfer function of the detailed GrC model for frequencies below 20 Hz, acquired using the “sinusoidal fit” method as explained later. The values obtained were *R* = 5227 MΩ (giving a time constant of τ = 15.7 ms) and spike output delay Δ_*s*_ = 4.85 ms. The fitted resonant current parameters were *g*_*b*_ = 55.6 pS (conductance increase when spike occurs) and τ_*b*_ = 19.6 ms (time constant).

Granule cells show a strong inward rectification with a nine-fold increase of resistance (D'Angelo et al., 1995) which is also reflected in the detailed model. Thus, while the resistance of the detailed model at rest (−71.5 mV) was 1049 MΩ, the resistance of the IF models was 5227 MΩ which reflects the effective lumped resistance around spike threshold.

Two stimulation modes, current and synaptic, were utilized to investigate the capabilities of these model for modulated firing rate (MFR) transmission.

### Current stimulation

Although the neuron's input can be a continuous variable (e.g., direct current injection), its spiking output is not continuous. In the following we try to avoid the ambiguity attached to the term frequency when discussing spiking codes by using the term firing rate (units spikes/s) rather than firing frequency to describe rate of spike production, and reserve the term frequency (units Hz) to describe the frequency of sinusoidal components of the continuous input signal.

For current stimulation, either a sinusoidally or stochastically modulated tonic excitatory current was injected into the model cells. This mode of stimulation allowed us to examine the contribution of the intrinsic conductance structure of the neuron to information processing. For both sinusoidal and stochastic modulations a tonic excitatory current *I*_0_ was chosen to produce a desired tonic firing-rate *F*_0_, which we refer to as the carrier-rate. The value of the carrier-rate was usually set to 40 spikes/s, a value that, according to the Nyquist theorem, in principle allows information transfer for modulation frequencies of up to 20 Hz corresponding to the upper frequency limit of natural primate head movements (see below).

For sinusoidal modulations *I*_*E*_ = *I*_0_ + *A*_*I*_ sin (2π*ft*) and for stochastic stimulations the excitatory current was *I*_*E*_ = *I*_0_ + *A*_*I*_ *x*(*t*) and the Gaussian process *x*(*t*) was normalized to 2σ = 1. The amplitude of modulation was then chosen so that a tonic input *I*_0_ + *A*_*I*_ produces a tonic firing rate (1 + *a*)*F*_0_, that is, a relative output modulation *a*. Since Gaussian processes are unbounded, this ensures that for stochastic stimulations with low amplitudes the interval (1 ± *a*)*F*_0_ includes the output firing rate approximately 95% of the time.

**Figure 1:**
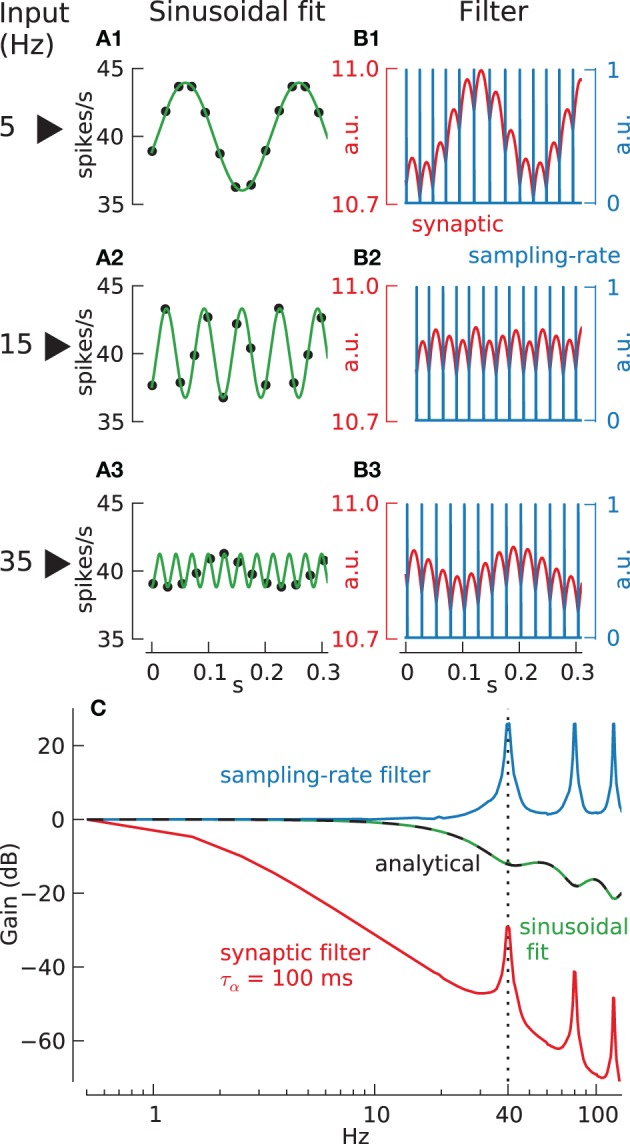
**Different methods to compute neuronal transfer function from spike times. (A)** Sinusoidal fit method: spike rate (black dots) and fitted sinusoids (green lines) for stimulation of integrate-and-fire neuron (τ = 15.7 ms) with current modulated at 5 Hz **(A1)**, 15 Hz **(A2)**, and 35 Hz **(A3)**. **(B)** Filter method: spike times, resulting from stimulation of integrate-and-fire neuron as in **(A)**, with current modulated at 5 Hz **(B1)**, 15 Hz **(B2)**, and 35 Hz **(B3)**, are convolved with either an alpha-function (with τ_α_ = 100 ms) (red lines), called “synaptic filter,” or a rectangular function (width = 0.025 ms, i.e., simulation step size) (blue lines), called “sampling-rate filter.” **(C)** Resulting transfer function using either sinusoidal fit (green line, analytical function: black dashed line) or filter methods (synaptic, red line; sampling-rate, blue line).

**Figure 2:**
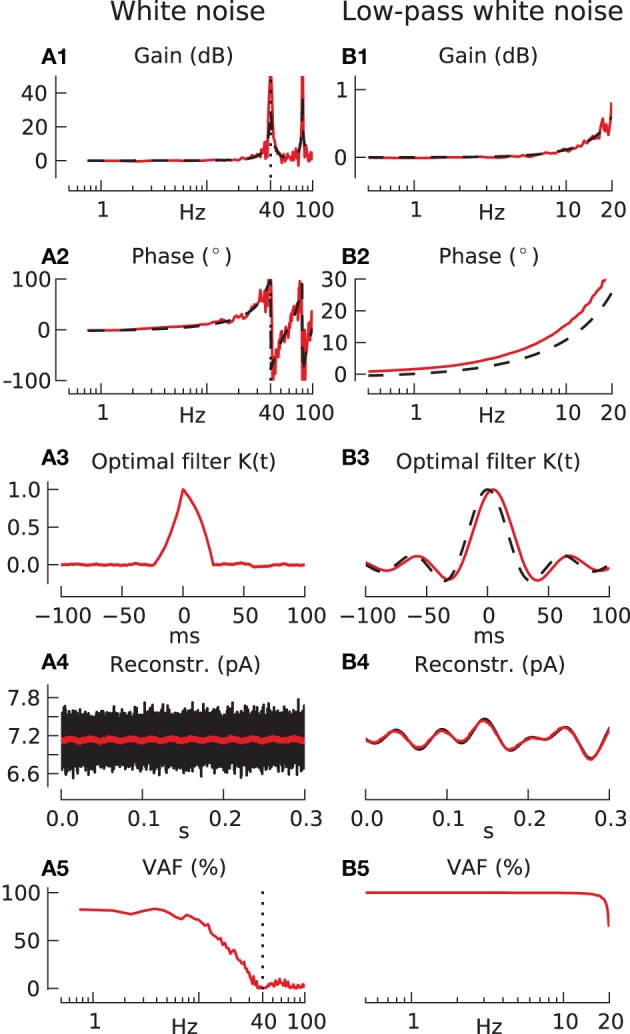
**Response of integrate-and-fire neuron to white noise stimulation using sampling-rate filter and ideal observer methods**. Parameters for all following simulations: carrier-rate *F*_0_ = 40 spikes/s, modulation amplitude *a* = 0.1. **(A)** Response to unfiltered white noise. **(A1,A2)** Show transfer function gain and phase respectively (red line), compared with analytically derived response (black dashed line). **(A3)** Shows the optimal filter for unfiltered white noise, **(A4)** the results of its use in a sample reconstruction (black shows input, red shows output), and **(A5)** the reconstruction quality (VAF). **(B)** Response to white noise low-pass filtered at 20 Hz. **(B1–B5)** as in **(A)**. The black dashed line in **(B3)** is the function *f*(*t*) = sinc (2π · 20 *Hz* · *t*), which is the Fourier transform of the 20 Hz white noise filter.

**Figure 3:**
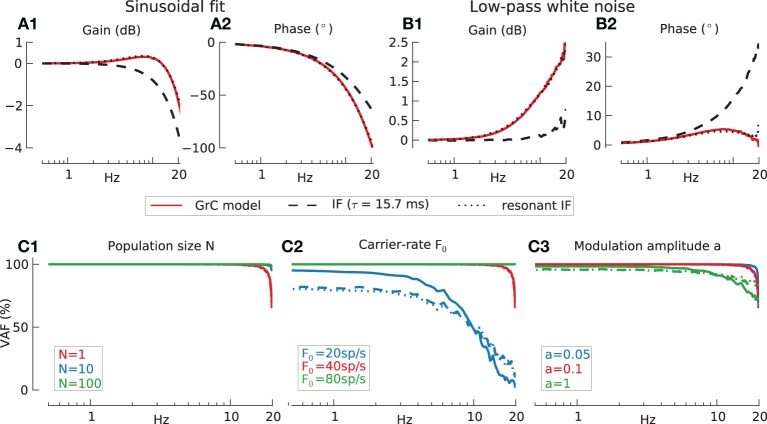
**Information transmission by model GrC in response to modulated current injection, assessed by two methods**. Baseline conditions for all following simulations: population size *N* = 1, carrier-rate *F*_0_ = 40 spikes/s, modulation amplitude *a* = 0.1. **(A)** Sinusoidal fitting. Transfer function gain **(A1)** and phase **(A2)** for detailed GrC model (red lines), passive integrate-and-fire (IF) neuron (dashed lines) and resonant integrate-and-fire neuron (rIF) (dotted lines) under current stimulation. **(B)** Sampling rate filter for 20 Hz low-pass filtered white noise. Transfer function gain **(B1)** and phase **(B2)** for three neuronal models (legend as in **A**). **(C)** Reconstruction quality (VAF) for different neuron models under varying conditions. Legend for all panels: solid line indicates the detailed GrC model, dashed line the passive integrate-and-fire neuron (IF) and dotted line the resonant integrate-and-fire neuron (rIF). **(C1)** Effects of population size (*N* = 1: red, *N* = 10: blue, and *N* = 100: green lines). Mean % VAF for *N* = 1 97.9 GrC; 97.8 IF; 98.1 rIF. For *N* = 10 99.8, 99.7, and 99.8 respectively. For *N* = 100 all 99.9. **(C2)** Effects of carrier-rate (20 spikes/s, blue: 40 spikes/s, red: 80 spikes/s green). Mean % VAF for 20 spikes/s 49.3 GrC; 49.2 IF; 49.4 rIF. For 40 spikes/s 97.9, 97.8, and 98.1 respectively. For 80 spikes/s all 100. **(C3)** Effects of modulation amplitude a (*a* = 0.05 blue, *a* = 0.1 red *a* = 1: green). Mean % VAF for *a* = 0.05 99.2 GrC; 99.0 IF; 99.2 rIF. For *a* = 0.1 97.9, 97.8, and 98.1 respectively. For *a* = 1 90.6, 91.3, and 89.2 respectively.

**Figure 4:**
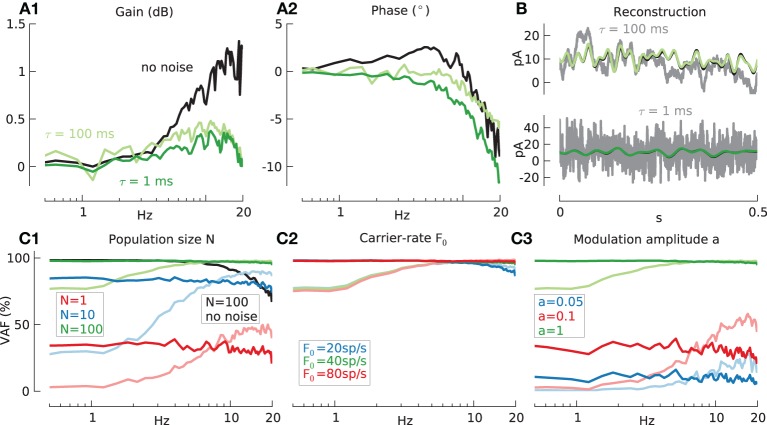
**Information transmission by model GrC in response to modulated current injection, with additive noise**. Baseline condition for all following simulations: population size *N* = 100, mean carrier-rate *m*(*F*_0_) ≈ 40 spikes/s, *std*(*F*_0_) ≈ 0 spikes/s and modulation amplitude a = 1. **(A)** Effects of noise on transfer function of GrC model **(A1** gain, **A2** phase) measured using the sampling-rate filter method, with 20 Hz low-pass filtered white noise as the input signal (black lines) and fast (τ_*n*_ = 1 ms, *a*_*n*_ = 4, dark green lines) or slow (τ_*n*_ = 100 ms, *a*_*n*_ = 2, light green lines) additive correlated white noise. **(B)** Reconstruction, from stimulation with additive fast (green line) or slow (light green line) noise, of input signal (black line) from GrC response. Total input current to one exemplary cell shown in gray. **(C)** Reconstruction quality (VAF) for GrC model under varying conditions. **(C1)** Effects of population size N and fast noise (*N* = 1: red, *N* = 10: blue, and *N* = 100: green line), slow noise (*N* = 1: light red, *N* = 10: light blue, and *N* = 100: light green line) or without noise (*N* = 100: black line). Mean % VAF for *N* = 1 32.1 fast noise; 33.6 slow noise. For *N* = 10 81.6 and 76.7 respectively. For *N* = 100 97.1 and 95.1 respectively. For *N* = 100 without noise 89.2. **(C2)** Effects of carrier-rate *F*_0_ and fast noise (20 spikes/s, blue: 40 spikes/s, red: 80 spikes/s green) or slow noise (20 spikes/s, light blue: 40 spikes/s, light red: 80 spikes/s light green). Mean % VAF for 20 spikes/s 95.0 fast noise; 93.4 slow noise. For 40 spikes/s 97.1 and 95.1 respectively. For 80 spikes/s 97.6 and 95.2 respectively. **(C3)** Effects of modulation amplitude a and fast noise (*a* = 0.05: blue, *a* = 0.1: red, and *a* = 1: green line) or slow noise (*a* = 0.05: light blue, *a* = 0.1: light red, and *a* = 1: light green lines) Mean % VAF for *a* = 0.05 10.0 fast noise; 13.6 slow noise. For *a* = 0.1 31.0 and 35.6 respectively. For *a* = 1 97.1 and 95.1 respectively.

In cases where the relative modulation amplitude *a* is not larger than one (Figures 1–**4**) the carrier-rate *F*_0_ is approximately equal to the effective firing-rate *F*_*eff*_ which was defined as the activity in presence of modulatory input.

### Measures of information transmission

Quantifying information transmission and transformation for spike-coded signals is in general a very difficult problem (Dayan and Abbott, 2001). We wish to emphasize here three important methodological considerations that must be taken into account when interpreting neural transfer functions. Firstly, neural outputs (and possibly inputs) are spiking, hence the estimated transfer function depends critically on the method used to transform spiking to continuous outputs. We will motivate here the use of a sampling-rate filter to obtain this estimate. Secondly, since a linear transfer function cannot represent the non-linear (and potentially noisy) spike generation process perfectly accurately, a transfer function estimate must be accompanied by an estimate of its fidelity. We will show that evaluation of a companion statistic such as variance-accounted-for (VAF), estimated with an ideal observer Wiener filter method (Gabbiani and Koch, 1998), is crucial in interpreting transfer function estimates. Finally, it is important to recognize that the information transfer performance of the neuron depends crucially on the statistics of the input signals required for a behavioral task; these must be specified as part of the analysis if the results are not to be misleading.

To illustrate the importance of this we consider the commonly used technique for transfer function estimation illustrated in Figure 1, where the neuron's response to sinusoidal current inputs is measured at a range of different frequencies, and the output gain and phase is plotted as a function of input frequency (the Bode plot). In this method a sinusoidal fit (with the frequency of the interpolated sinusoid taken to be the known input modulation frequency) to the instantaneous firing frequency (calculated from inter-spike-intervals) is used to estimate a continuous neural output. Panels A1–A3 show the response of a passive integrate-and-fire (IF) neuron to sinusoidal modulated direct current stimulation. In the example shown the carrier-rate is *F*_0_ = 40 spikes/s the relative modulation amplitude is *a* = 0.1 and the sinusoidal modulation frequencies are *f* = 5, 15 and 35 Hz.

The first column shows the best sinusoidal fit through the cell's instantaneous firing rate against spike time (filled circles). Comparison of input and output modulation amplitudes gives the transfer function shown in the bottom panel Figure 1C (green line marked sinusoid fit) which can also be calculated analytically (black dashed line) (Knight, 1972). For frequencies below the carrier-rate of 40 spikes/s the neuron's gain declines monotonically above ~10 Hz, whereas above the carrier-rate there are resonance lobes in the transfer function at particular frequencies. These resonances are caused by “locking,” a phenomenon in which spikes are entrained preferentially into a particular phase relationship to the underling signal.

The transfer function obtained in this way seems to demonstrate substantial information transmission above 20 Hz. However, inspection of the spiking output reveals that the particular form taken by the transfer function at these high frequencies depends critically upon our knowing what frequency of sinusoid to fit to the discrete firing rate data. The plotted sinusoidal fits (green curves) show that at low frequencies the fit accurately reflects the behavior of the sampled data (5, 15 Hz in Figures 1A1,A2). However, at frequencies above the Nyquist frequency of 20 Hz (35 Hz, Figure 1A3) this is no longer the case. For example it is unlikely that the 35 Hz sinusoid shown (green line) would have been the preferred reconstruction of the data in Figure 1A3 without prior knowledge of the input frequency, and since this prior knowledge could not be available in any behaving system this particular reconstruction must be considered artificial.

This explains an apparent conflict between Figure 1C and the Shannon sampling theorem. Since a single neuron can only transfer information at the times when spikes occur, its output is effectively sampled at an average rate given by the carrier-rate. Hence by the Shannon sampling theorem the frequency content beyond the Nyquist frequency (half the carrier-rate) is not well-defined and we have unambiguous information transfer only below this frequency. It is our prior knowledge of the input frequency that apparently allows this limit to be transcended. Hence, although the Bode plot in Figure 1C (green line) accurately represents the results of a particular kind of experiment, the transfer function is misleading, since it suggest that information transfer by a single neuron employing MFR coding is not limited by the Nyquist frequency. To investigate this limitation further we must consider other decoding methods.

#### Direct estimation of transfer functions: the sampling-rate filter

A more relevant method for transforming neuronal spiking output into a continuous variable is to apply an appropriate linear filter. This avoids the need for prior assumptions, as in sinusoidal fitting, and it approximates the physical conversion of spikes into postsynaptic potential changes. One problem is that the transfer function obtained using this method depends on the filter chosen. For example Column B of Figure 1 shows the response using two different types of filter. The red curve shows the output of the IF neuron passed through a model synaptic filter (alpha-function, low-pass filter, with time constant 100 ms) The blue curve shows the output passed through a delta-function filter, approximated in discrete time as a sampling-rate filter (a rectangular window filter with width equal to the simulation step size *dt* = 0.025 ms) so that each time a spike occurs the output function is set to 1 and 0 otherwise. Subsequently, the corresponding normalized transfer function gain (Figure 1C, blue and red lines) can be derived directly from the continuous output *y*(*t*) (Figures 1B1–B3, red and blue lines) and input data *x*(*t*) by dividing their respective Fourier-amplitudes at corresponding sinusoid input frequencies: *mag* = [|*FT*(*x*(*t*),*f*)|/|*FT*(*y*(*t*),*f*)|]^200 *Hz*^_*f* = 0.5 *Hz*_.

Clearly a central question for this method is which filter to choose. Since we do not wish to limit *a-priori* the efficiency of subsequent processing we propose a “direct estimation” of the transfer function, i.e., employ the sampling-rate filter but use variance-accounted-for (VAF) statistics, estimated with an ideal observer Wiener filter method (Gabbiani and Koch, 1998), to interpret the signal transmission fidelity.

#### The ideal observer

The Wiener filter that allows the most accurate subsequent linear reconstruction of the input signal is termed the non-causal ideal observer (Gabbiani and Koch, 1998) and has been applied, for example, to quantify the accuracy of stimulus encoding in vestibular afferents (Sadeghi et al., 2007). A requirement for this method is that the input now has to be changed from single sinusoids to a Gaussian process. For input signals *x*(*t*) and spiking (delta-function sampling-rate filter) outputs *y*(*t*) the transfer function estimate is

(2) $$T(f)=\frac{P_{xy}(f)}{P_{xx}(f)}$$

and the optimal reconstruction filter is:

(3) $$K(f)=\frac{P_{yx}(f)}{P_{yy}(f)}$$

where *P*_*xx*_, *P*_*yy*_, *P*_*xy*_, *P*_*yx*_ are the spectral and cross-spectral densities of the processes *x* and *y*, computed using Welch's average periodogram method (Bendat and Piersol, 2010). The optimal reconstructed input is *x*_est_ = *K* * *y* and the accuracy of reconstruction can be assessed using the variance-accounted-for statistic:

(4) $$VAF(f)=\frac{{\left|P_{xy}(f)\right|}^{2}}{P_{xx}(f)P_{yy}(f)}$$

which is usually expressed as a percentage, so that *VAF*(*f*) = 100% implies perfect reconstruction at that frequency and thus a high fidelity in signal transmission.

Although use of the ideal observer eliminates the dependence on the choice of reconstruction filter, it introduces a new dependence on the statistics of the input process as illustrated in Figure 2. In Column A of this figure the input process is chosen to be a completely unpredictable white noise process. The numerically estimated transfer function in panels A1, A2 (red lines) shows signal transmission at all frequencies with a regular series of infinite resonance peaks (the analytical transfer function is also known in this case (Knight, 1972) and is shown for comparison as black dashed line). However, these prominent features of the Bode plot do not indicate significant information transfer at high frequencies. Applying the optimal reconstruction filter (which can be seen to be an approximate triangular function at the spike sampling time scale, shown in panel A3) reveals a complete failure to reconstruct (red plot in A4) any high frequency detail in the white noise input (black plot). This failure is predicted by the VAF plot (A5) which shows unsatisfactory reconstruction even at low frequencies (maximum VAF ~ 75%) and a falloff in VAF around the Nyquist frequency (20 Hz) with no useful reconstruction beyond the carrier-rate of 40 spikes/s.

In column B the input is taken to be band-limited white noise with a flat spectrum up to a cutoff frequency of 20 Hz equal to the Nyquist frequency. The transfer function is plotted in panels B1, B2 only up to the Nyquist frequency, since higher frequencies are not present in the input. Up to this limit it is identical to that in column A. The ideal reconstruction filter shown in panel B3, it is essentially a sinc (band-pass reconstruction) function, and a sample input reconstruction is shown in panel B4. The reconstructed (red) signal accurately overlays the band-passed input (black), and this accuracy is predicted in the VAF plot in panel B5, which shows almost perfect reconstruction, VAF ~ 100%, all the way up to the Nyquist frequency. Using colored noise inputs between these two extremes of white and band-passed white noise (results not shown) gives intermediate results, with some information transmission possible at higher frequencies and subsequent information loss at low frequencies.

Clearly we need to choose a suitable candidate for input-signal process statistics. Although white noise signals are important theoretically they are very unlike the usual signals found in sensorimotor systems and transmitted by MFR coding. For example, natural head movements in people and monkeys have most of their power below 20 Hz (see Discussion). For this reason we have chosen band-passed white noise inputs with a low-pass frequency cutoff of 20 Hz for most of the simulations in this paper. However, since the realistic limit for cerebellar cortical involvement in vestibular processing may be lower, we will also test for cutoffs at lower frequencies.

While the loss of fidelity in the above simulations is produced by the spike-sampling process alone, i.e., converting the continuous input signal to a signal represented by spike events, we also analyzed signal transmission properties under the influence of a separate source of input noise modeled as an additive Ornstein-Uhlenbeck stochastic process *n* (Destexhe et al., 2001) with a time constant of τ_*n*_ = 1 ms (fast noise) and τ_*n*_ = 100 ms (slow noise). The relative amplitude of the noise current *A*_*IN*_ = *a*_*n*_ · *F*_0_ was exemplarily set to *a*_*n*_ = 4 for fast and *a*_*n*_ = 2 for slow noise.

#### Population coding

The methodology described above can also be applied to input reconstruction from the population spiking output. Analysis of white noise signal transmission with small carrier-rate and large population sizes was carried out for an example population of 100 detailed GrCs, resonant and passive IF models with and without slow current noise (τ_*n*_ = 100 ms) as above.

Since in simulations with low firing-rate and in following synaptic simulations the carrier-rate *F*_0_ was not equal to the effective firing-rate *F*_*eff*_ we focused on the estimation of the latter since we considered it to be a more truthful value to compare different models and configurations. The modulation amplitude was thus set to *A*_*I*_ = 2 pA for all cases, whereas the noise amplitude *A*_*IN*_ and the mean and standard deviation of the tonic subthreshold stimulation current *I*_0_ across the population was adjusted to result in an effective population firing-rate of mean(*F*_*eff*_) = 4 spikes/s and *std*(*F*_*eff*_) = 2 spikes/s for all cells and configurations.

A second coding scheme for populations, termed “push-pull coding,” was employed where half of the neurons constitute a second sub-population that receive the inverted signal −1 · x(*t*) and the output to be reconstructed, used for transfer function and VAF computations, is *y*(*t*) = *y*_1_(*t*) − *y*_2_(*t*) (Bialek et al., 1991).

### Synaptic stimulation

The second stimulation mode was synaptic, via stochastic modulation of the firing rate of the excitatory and inhibitory inputs about a tonic rate; this mode allowed the additional contribution from details of synaptic processing to be examined. Each model cell had 4 excitatory synapses (AMPA, NMDA) and 4 inhibitory synapses (GABA) reflecting realistic convergence ratios (Solinas et al., 2010). Each excitatory synapse received a representation of the input signal *R*(*t*) = *a* · *F*_0*in*_ · *x*(*t*) + *F*_0*in*_ with a different input carrier-rate *F*_0*in*_ chosen with mean of 40 spikes/s (reflecting realistic value as seen for vestibular mossy fibers, Lisberger and Fuchs, 1978) and standard deviation of 10 spikes/s so that 95% of input spike-rates lay between 20 and 60 spikes/s, e.g., 2 · *std*(*F*_0*in*_) = *v* · *m*(*F*_0*in*_) with a relative variance *v* = 0.5. In some cases *v* was increased to 0.7 or 1.2. The Gaussian process *x*(*t*) was normalized to 2σ = 1 which ensures that the amplitude *a* · *F*_0*in*_ includes the input firing rate 95% of the time. Each inhibitory synapse normally received either the same constant frequency input *F*_*I*_, or normally distributed carrier-rates with 2 · *std*(*F*_*I*_) = 0.5 · *m*(*F*_*I*_). In another simulation stochastically modulated signals were also sent through the inhibitory synapses with *R*(*t*) = *a* · *F*_*I*_ · *x*(*t*) + *F*_*I*_.

To generate spiking inputs from the firing rate *R*(*t*) we require a standard method for generating an input spike train from a continuous input signal. Spikes were generated using an ideal (τ = ∞) integrate-and-fire neuron with resting and reset potential 0 and spike threshold 1 (Knight, 1972) which was chosen because of its flat transfer function (see Results). To achieve identical *F*_*eff*_ and identical *F*_*I*_ but different input modulations or cutoff frequencies, the spike rate was additionally controlled by a constant inhibitory current *I*_*I*_ = *g*_*I*_(*V* − 65 mV) in some simulations.

Push-pull coding was also tested using synaptic stimulation. Here all 4 synapses of 50% of the population received either the normal or the inverted signal −1 · *x*(*t*).

### General notes

Gain is always normalized to unity at the lowest frequency and plotted in decibel (dB) with mag_dB_ = 20 log_10_(mag) and phase is always measured in degree (°). For all simulations a VAF of 90% is used as a threshold for desired coding quality.

**Figure 5:**
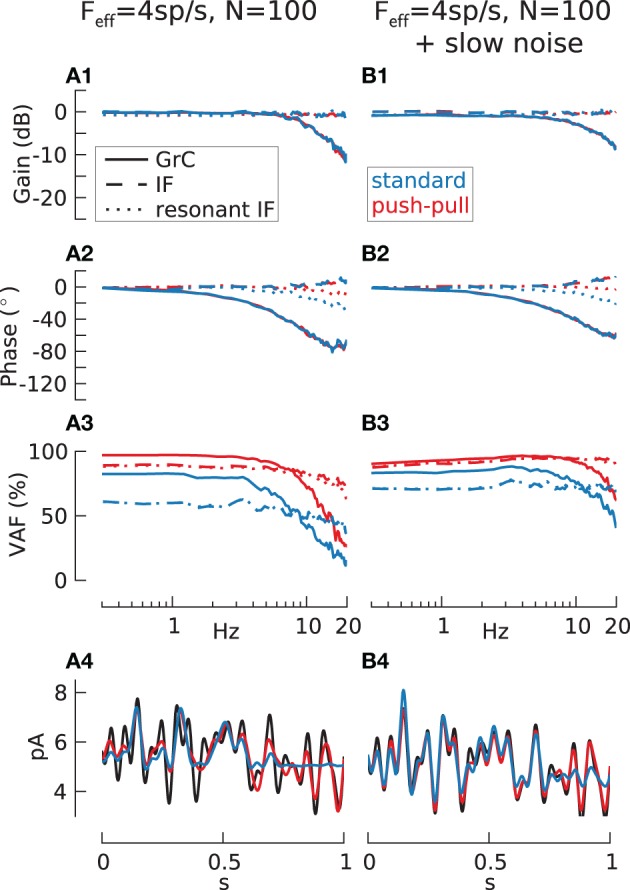
**Transmission with low firing-rate**. Transfer function gain **(A1,B1)**, phase **(A2,B2)**, VAF **(A3,B3)** and reconstruction sample **(A4,B4)** for 100 cell populations of passive IF (dashed lines), resonant IF (rIF) (dotted lines) and GrC model (solid lines) without **(A)** and with **(B)** additive filtered slow noise (τ_*n*_ = 100 ms). In addition to the normal case where population is encoding the whole signal (blue lines) push-pull coding (red lines) is shown. In this case, half of the cells encode only the positive and the negative part of the signal respectively. Input current adjusted for all recordings to result in *m*(*F*_*eff*_) = 4 spikes/s and *std*(*F*_*eff*_) = 2 spikes/s. [*m*(*VAF*) in % for IF/rIF/GrC: 50.9/50.8/46.4; 82.5/80.1/68.3 (push-pull); 72.0/73.1/72.4 (slow noise); 93.8/93.1/87.6 (push-pull + slow noise)].

**Figure 6:**
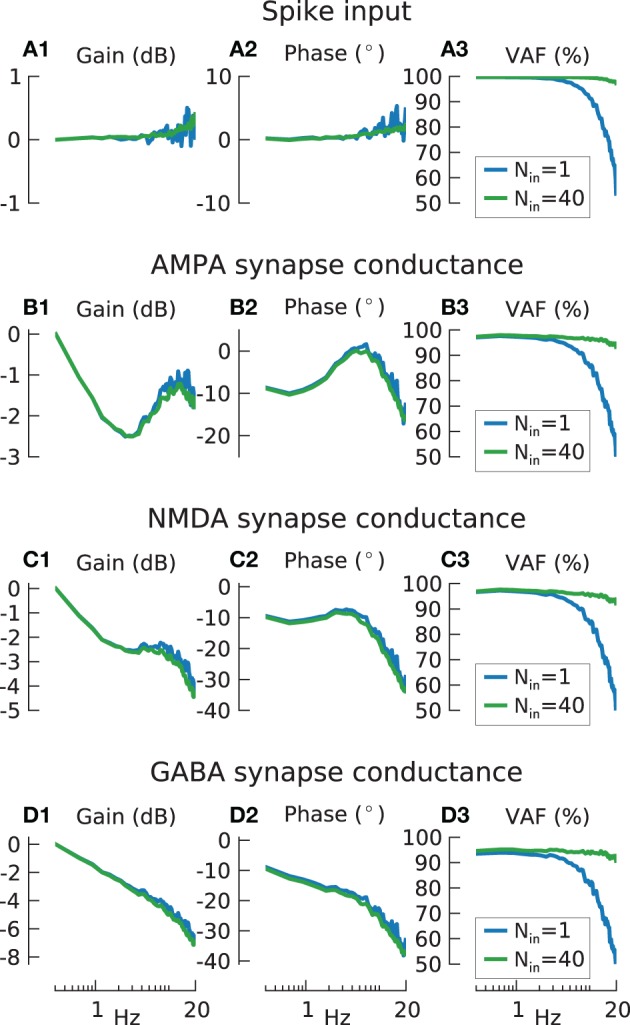
**Information transmission by input spike train encoder and synaptic conductances. (A)** Transfer function gain **(A1)** and phase **(A2)** for input spike trains produced by ideal integrate-and-fire (IF) neurons (blue lines *N* = 1, green lines *N*_*in*_ = 40), with input carrier-rate *F*_0*in*_ = 40 spikes/s and modulation amplitude *a* = 1 stimulated by 20 Hz low-pass filtered white noise. Gain values are normalized to the lowest frequency for the results from *N* = 1. **(A3)** shows variance accounted for. The mean % VAF is 85.9 for *N* = 1, 99.1 for *N* = 40. **(B)** As in **(A)**, but with the spike-train output of the ideal IF neurons additionally passed through model AMPA synapses. The mean VAF is 80.7 for *N* = 1, 96.1 for *N* = 40. **(C)** As in **(B)**, but for model NMDA synapses. The mean % VAF is 79.8 for *N* = 1, 95.3 for *N* = 40. **(D)** As in **(B)**, but for model GABA synapses. The mean % VAF is 76.9 for *N* = 1, 93.7 for *N* = 40.

**Figure 7:**
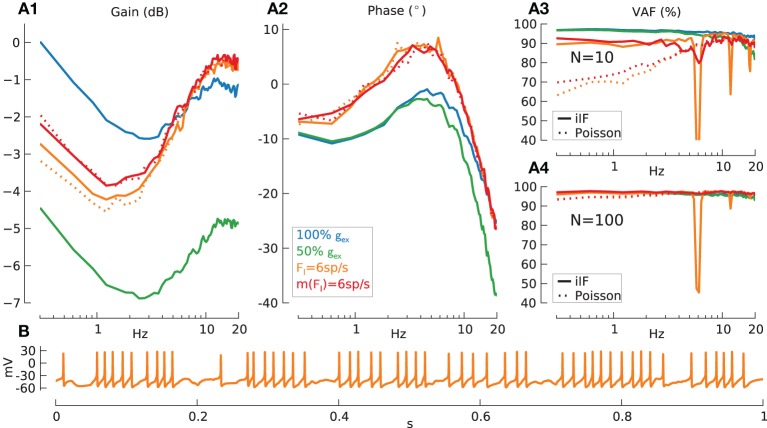
**Information transmission for synaptic activation (AMPA + NMDA) and inhibition (GABA) on signal transmission**. Mean, standard deviation of effective firing-rate in spikes/s and mean % VAF given as triplet [*m*(*F*_*eff*_)/*std*(*F*_*eff*_)/*m*(*VAF*)] in the following. Baseline condition for all following simulations: modulation amplitude *a* = 1. **(A)** Gain **(A1)**, phase **(A2)**, and reconstruction quality (VAF) **(A3)** for *N* = 10 GrC model cells and VAF for *N* = 100 GrC model cells **(A4)** with following conditions: without inhibition but standard synaptic strength [blue lines, *N* = 10: **(A1–A3)** (109/6.8/95.2), *N* = 100: **(A4)** (111/6.9/96.3)], without inhibition and reduced (50%) synaptic conductance [green lines, *N* = 10: **(A1–A3)** (39/4.1/92.2), *N* = 100: **(A4)** (40/4.1/95.6)], with constant inhibition *F*_*I*_ = 6 spikes/s [orange lines, *N* = 10: **(A1–A3)** (36/5.5/89.4), *N* = 100: **(A4)** (37/5.5/95.1)] and variable *m*(*F*_*I*_) = 6 spikes/s inhibition [red lines, *N* = 10: **(A1–A3)** (39/8.2/90.1), *N* = 100: **(A4)** (38.2/7.8/96.9)]. Input spike train encoded using iIF (τ = ∞) (solid lines). Furthermore, results with inhibition coded using inhomogeneous Poisson process (dotted lines): constant inhibition *F*_*I*_ = 6 spikes/s [dotted orange lines, *N* = 10: **(A1–A3)** (37/5.4/87.6), *N* = 100: **(A4)** (38/5.4/96.6)] and variable inhibition *m*(*F*_*I*_) = 6 spikes/s [dotted red lines, *N* = 10: **(A1–A3)** (40/8/88.3), *N* = 100: **(A4)** (39/7.6/96.7)]. Gain was normalized to the lowest frequency for the results from *F*_*I*_ = 0 spikes/s and full synaptic conductance (blue solid line). **(B)** Membrane potential trace of respective GrC model cell in **(A)** with non-variable inhibition (*F*_*I*_ = 6 spikes/s).

**Figure 8:**
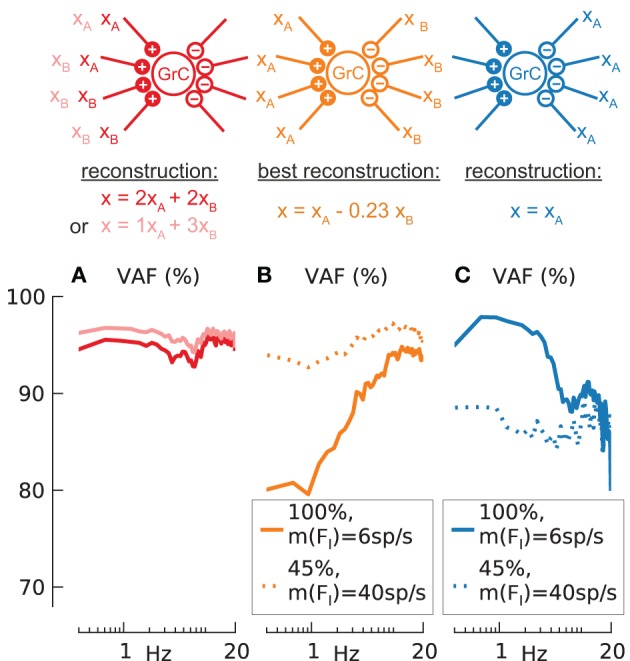
**Synaptic signal combination and signal trough inhibitory synapse**. Reconstruction quality (VAF) **(A–C)** of *N* = 100 synaptically activated (AMPA + NMDA) granule cells with *m*(*F*_*I*_) = 6 spikes/s but different input configurations as depicted by clipart above. Mean, standard deviation of effective firing-rate in spikes/s and mean of VAF in % given as triplet [*m*(*F*_*eff*_)/*std*(*F*_*eff*_)/*m*(*VAF*)] in the following. Baseline condition for all following simulations: modulation amplitude *a* = 1. **(A)** Two different input signals to two excitatory synapses respectively (dark red line) (37/8.2/95.0) or to one and three excitatory synapses (light red line) (37/8.0/95.9). **(B)** Two different input signals to 4 excitatory and 4 inhibitory synapses respectively, best reconstruction *x*(*t*) = *x*_A_(*t*) −0.23*x*_*B*_(*t*) (solid orange line) (38/7.7/91.9). Additional simulations for reduced synaptic conductance (45%) and increased inhibition rate *m*(*F*_*I*_) = 40 spikes/s (dotted orange line) (38/6.3/96.1). **(C)** One input signal to the 4 inhibitory synapses only (solid blue line) (38/8.6/90.2). Additional simulations for reduced synaptic conductance (45%) and increased inhibition rate *m*(*F*_*I*_) = 40 spikes/s, (dotted blue line) (38/7.2/86.6).

**Figure 9:**
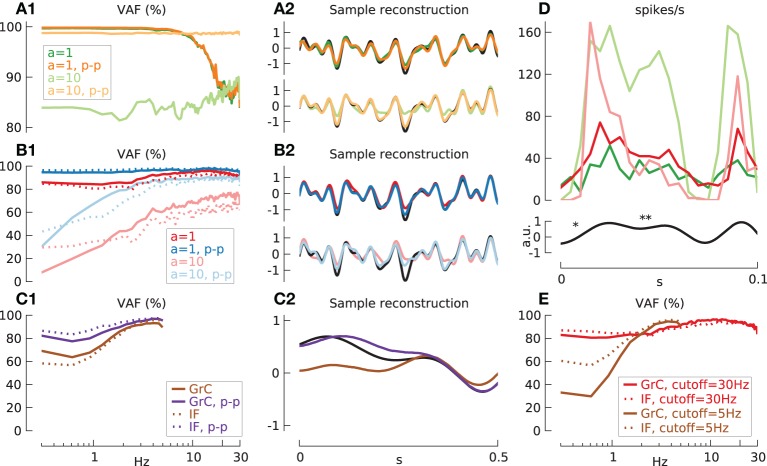
**Transmission quality depends on modulation amplitude a, maximum low-pass filtered noise input frequency (cutoff) and push-pull coding. (A)** Reconstruction quality (VAF) **(A1,B1,C1)** and sample reconstruction **(A2,B2,C2)** with *N* = 100. Mean, standard deviation of effective firing-rate in spikes/s and mean of VAF in % given as triplet [*m*(*F*_*eff*_)/*std*(*F*_*eff*_)/*m*(*VAF*)] in the following. **(A)** iIF input with cutoff = 30 Hz and *F*_0*in*_ = 20 spikes/s: *a* = 1, green lines (19.5/5.0/92.9); *a* = 10, light green lines (44.5/10.6/85.7); *a* = 1, push-pull, orange lines (19.5/5.0/93.2); *a* = 10, push-pull, light orange lines (44.5/10.6/98.7). **(B)** Input signal (cutoff = 30 Hz) through synaptic excitation, *m*(*F*_0*in*_) = 40 spikes/s and additional inhibition of *m*(*F*_*I*_) = 9 spikes/s. **(B1,B2)**: GrC model: *a* = 1: red lines, (21.8/6.6/93.0); push-pull: blue lines (21.1/6.4/96.5). *a* = 10, *g*_*I*_ = 0.14 nS: light red lines (21.1/4.5/67.4); push-pull: light blue lines (20.6/4.4/87.5). **(B1)** IF cell: *a* = 1, *g*_*I*_ = 0.19 nS: dotted red line (21.5/8.6/90.7); push-pull: dotted blue line (20.7/8.6/97.6). *a* = 10, *g*_*I*_ = 0.4 nS: dotted light red line (21.4/6.0/59.1); push-pull: dotted light blue lines (20.8/6.0/84.5). **(C)** Input (cutoff = 5 Hz) through synaptic excitation, *m*(*F*_0*in*_) = 40 spikes/s, *a* = 1 and low firing-rate due to increased inhibition of *m*(*F*_*I*_) = 14 spikes/s. **(C1,C2**) GrC model: brown lines (3.6/3.4/84.9); push-pull: purple lines (3.3/3.2/90.9). **(C1)** IF model, *g*_*I*_ = 0.19 nS: dotted brown line (3.7/3.2/84.2); push-pull: dotted purple line (3.4/2.9/93.6). **(D)** Sample spike-rate (binsize 5 ms) of 100 ideal IF cells (*a* = 1, green line and *a* = 10, light green line) and 100 synaptically activated GrC models (*a* = 1, red line and *a* = 10, light red line) in response to short 30 Hz low-pass filtered white noise input (black line) with high- (^*^) and low frequency components (^**^). **(E)** Excitatory synaptic stimulation [*m*(*F*_0*in*_) = 40 spikes/s, *a* = 1] with constant inhibitory current *I*_*I*_ only. High *F*_*eff*_, cutoff = 30 Hz: GrC model, *g*_*I*_ = 0.56 nS and increased relative variance *v* = 0.7: red line, (20.1/6.7/92.3); IF model, *g*_*I*_ = 0.74 nS: dotted red line, (20.5/8.9/91.3). Low *F*_*eff*_, cutoff = 5 Hz and *v* = 1.4: GrC model, *g*_*I*_ = 0.635 nS: brown line, (3.3/3.1/78.5); IF model, *g*_*I*_ = 0.84 nS: dotted brown line, (3.3/3.1/85.0).

**Figure 10:**
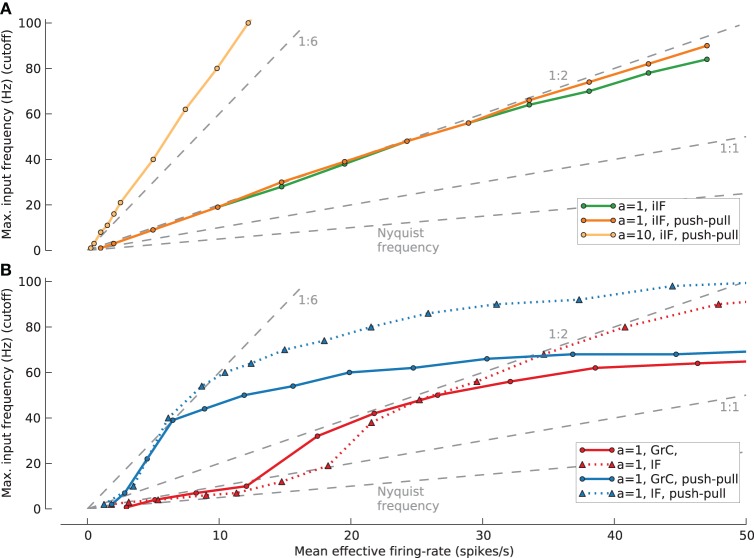
**Signal with maximum input frequency (cutoff) that can be transmitted with a mean reconstruction quality VAF > 90% for populations of N = 100 under different firing-rates (*F*_*eff*_). (A)** Results for ideal IF cell (iIF) (green line/circles: *a* = 1; orange line/circles: *a* = 1, push-pull coding; light orange line/circles: *a* = 10, push-pull coding). **(B)** Results for synaptically stimulated GrC population (red line/circles: *a* = 1; blue line/circles: *a* = 1, push-pull) and IF population (red dotted line/triangles: *a* = 1; blue dotted line/traingles: *a* = 1, push-pull coding). Different levels of firing-rates for GrC and IF resulting from increased mean inhibition rate (*F*_*I*_). Back dashed lines give markers for different cutoff:*F*_*eff*_ relations from 2:1 (Nyquist frequency) to 1:6.

The carrier-rate *F*_0_, which is the activity without modulatory input, is in general not equal to the effective firing-rate *F*_*eff*_, which is the activity during modulatory input. They both are approximately equal during suprathreshold tonic current injections with *a*≤ 1 (Figures 1–**4**). In cases with subthreshold current (**Figure 5**) or synaptic stimulation (**Figures 6–10**) we considered the effective-firing rate *F*_*eff*_ to be a more truthful value to compare different models and configurations. For comparison, we estimated *F*_0_ from the spike activity without modulatory input in some of these simulations.

## Results

This section first describes signal transmission by model granule cells (GrCs) in response to simulated injections of current. We first compare our method of direct transfer function estimation to the sinusoidal fit method and analyse the effect of different properties on the fidelity. Furthermore, we explore the influence of noise and low carrier-rates. Subsequently we analyse the signal transmission and linearity during synaptic stimulation of excitatory and inhibitory synapses.

### Current injection

Current stimulation allowed us to examine the contribution of the intrinsic electro-responsive properties of model granule cells to signal processing.

#### Noise-free stimulation

We looked first at granule-cell firing rate produced by sinusoidal modulation of a steady current. Transfer functions were derived either by using sinusoidal fitting (Figure 3A), or using the direct estimation with a sampling-rate filter (Figure 3B) (see Materials and Methods for details). The red Bode plots in A1,A2, B1,B2 refer to the Solinas et al. (2010) model of the granule cell (GrC). Bode plots for the best fit passive integrate-and-fire (IF) neuron (dashed black line) and a resonant IF (rIF) neuron (dotted black line) are also shown. As explained in the Materials and Methods Section, the steady stimulation current gives a carrier-rate of ~40 spikes/s and plots are only shown up to the Nyquist frequency of 20 Hz. The best-fit IF model is unable to accurately reproduce significant features of the model GrC transfer function. It does not show the small resonance peak (panel A1) found using the sinusoidal-fit method, and it underestimates the size of the associated phase-lag (panel A2). Using the sampling-rate method the best-fit IF model underestimates the gain increase as the Nyquist frequency is approached (panel B1) and overestimates the phase lead (panel B2). However, these features of the GrC model could be accurately fitted (dotted lines) by extending the model to a resonant IF neuron with a spike delay (Materials and Methods) resulting in a peak resonance of 10 Hz. The spike delay had to be added to explain the phase lag [Figures 3A2,B2; compare phase of GrC model (red line) to passive IF model (dashed black line)], that cannot be produced by the resonant current and reflects the delay induced by ion-channel spike generation.

More informative than the transfer function, however, is a measure of how well the input stimuli are reconstructed using a non-causal ideal linear observer (Materials and Methods). Figure 3C shows the percentage variance-accounted-for (VAF) for the passive IF (dashed), resonant IF (dotted), and the detailed GrC model (solid lines). All models showed excellent stimulus reconstruction up to 20 Hz even for a population size of one (Figure 3C1) (the overlap in performance between the different models means the plots for individual models cannot be distinguished). Thus, a single model neuron is capable of encoding signals over the input frequency range of natural head movements.

The dependence of VAF on carrier-rate for a single neuron is shown in Figure 3C2. As the Shannon criterion suggests the minimum carrier-rate required for faithful transmission of band-passed white noise is twice the maximum frequency in the signal to be transmitted. Due to the relative definition of modulation amplitude the effective amplitude increases with carrier-rate. To show that this factor is not the cause for the increase in fidelity in Figure 3C2 the simulations were validated with identical absolute modulation amplitudes of *aF*_0_ = 2 Hz for all carrier-rates resulting in the same increase of VAF with carrier-rate (results not shown).

The effect of modulation amplitude is further investigated in Figure 3C3. Here, in the absence of additive noise the linear transfer function approximation is most accurate at low modulation amplitudes as we would expect, but is acceptable up to quite large modulations. Even a modulation depth of *a* = 1, where the input occasionally drives the cell below its firing threshold, inducing signal rectification and decreasing spike resolution just above threshold, only leads to a moderate reduction in VAF. Since the relative amplitude *a* was never larger than 1, the carrier-rate *F*_0_ which is the activity without modulatory input was always approximately equal to the effective firing-rate *F*_*eff*_ which was defined as the spike activity during modulatory input.

From here on we will only use direct estimation (sampling-rate filter) and ideal linear observer method for transfer function and VAF calculations, respectively.

#### Effects of noise

Many neurons (for example those in cerebral cortex) receive a continuous barrage of inputs from many synapses (Brunel et al., 2001), which acts as a main source of noise. While granule cells have many fewer inputs the main source of noise for them is probably from stochastic vescicle release and neurotransmitter diffusion from other synapses in the glomeruli (DiGregorio et al., 2002; Mapelli et al., 2009). We therefore investigated the effects on signal transmission by the GrC model under the influence of either slow noise, mimicking neurotransmitter diffusion, by introducing additive white noise filtered by a time constant of τ_*n*_ = 100 ms or fast noise, mimicking stochastic vescicle release, with a time constant of τ_*n*_ = 1 ms.

As in the noise-free case, the input signal was white noise low-pass filtered with a cutoff at 20 Hz and the carrier-rate was set to 40 spikes/s. The default modulation amplitude however was increased from *a* = 0.1 to *a* = 1 and population size N was increased from *N* = 1 to *N* = 100 to yield cleaner transfer function plots.

The effects of noise on the transfer function of the model GrC cell are shown in panels 4A1,A2. Comparison of the transfer functions in the noise–free case (black lines) with those previously shown (Figures 3B1,B2; red lines) indicate that increasing amplitude modulation leads to a gain decrease in the absence of noise, and is accompanied by a drop in VAF for high frequencies (Figure 4C1; black lines) as shown before (Figure 3C3). Panels 4A1,A2 also show that the addition of filtered white noise (green lines) irrespective of slow or fast, exposes the spike resonance (Figure 3A1) which otherwise is hidden under the much larger carrier-rate resonance.

Figure 4B shows an example of the reconstruction (green lines) of the low-pass filtered white noise signal (black line) in the presence of slow noise (upper panel) or fast noise (lower panel) for population size *N* = 100. For comparison, in each panel the input current to an example single cell is shown in gray. Similar good performance is shown in both cases.

Figure 4C shows the effects of noise on the VAF measure for different model variables. The VAF is badly affected by noise for small numbers of cells. For *N* = 100 cells, however, the addition of noise actually has a beneficial effect and leads to an increase in mean VAF from 89.2% without noise (black lines) to 97.1% for fast noise (green lines) and 95.1% for slow noise (light green lines). A similar beneficial effect on the fidelity can be observed by a heterogeneous population carrier-rate. E.g., increasing the standard deviation of the carrier-rates in all cells from *std*(*F*_0_) = 0 spikes/s to *std*(*F*_0_) = 2 spikes/s increases the mean VAF to 96.8% (results not shown).

Further results were that the influence of the carrier-rate was low due to the large population size of *N* = 100 (Figure 4C2) and that the VAF also strongly depends on the modulation amplitude (Figure 4C3) due to increased signal-to-noise ratio (Gabbiani and Koch, 1998). Here the effect of increased amplitude, in contrast to the noiseless case (Figure 3C3), outweighs the disadvantages of occasional signal rectification.

The biggest difference between slow and fast additive noise is that the fast noise acts uniformly on the whole frequency range while slow noise leads to a decreased VAF preferentially at low frequencies. The fidelity of both IF models, using variables as in Figure 4C, was very similar to the GrC model (data not shown).

#### Low firing-rate with current stimulation

So far we have shown that lowering the carrier-rate has adverse effects on the transmission quality for a single cell (*N* = 1) (Figure 3C2, blue line). This lowered carrier-rate can however be offset by increasing the population size, e.g., *N* = 100 (Figure 4C2, blue line). To further analyse this effect we have simulated large populations with a firing-rate well below the Nyquist frequency in the following.

While in simulations before, the carrier-rate *F*_0_ was approximately equal to the effective firing-rate *F*_*eff*_ this now no longer is the case. In all following simulations different models and configurations are thus compared based on the effective-firing rate *F*_*eff*_.

While the intrinsic properties of the GrC model have only a slight influence on transmission properties for sufficiently high firing-rates, this ceases to be the case when the firing-rates are smaller than the maximum input signal frequency. When analysing all three models (GrC, resonant IF and passive IF) in a population of 100 cells with *m*(*F*_*eff*_) = 4 spikes/s (Figure 5A, blue lines) it can be observed that the gain and phase for the passive IF population is flat over the whole frequency range and the resonant IF population just shows a small phase lag at higher frequencies due to the induced spike delay (Figures 5A1,A2). In contrast, the response of the GrC model population shows a large gain decay and phase lag at higher frequencies. Similarly, the VAF (Figure 5A3) of the IF populations is rather flat between 40 and 60% over the whole frequency range, whereas for the GrC population, VAF falls off to lower values for 20 Hz which was already seen for the case *F*_0_ = 20 spikes/s (Figure 3C2). This relation can be explained by the effect that since the higher frequency content is highly damped, as seen from the strong gain decay, and therefore any reconstruction potential erroneous, the Wiener filter tries instead to optimize the low frequency content of the signal.

For all three models however, the VAF is too small to get a good signal reconstruction as shown for the GrC population (Figure 5A4, blue line). Due to the low firing-rate, only strong and persistent positive amplitudes result in a spike output and therefore can be reconstructed. One possible way to improve this is to use push-pull coding where two populations of the same cell type encode the positive and the negative amplitude respectively. While this can indeed increase the VAF (Figure 5A3, red lines) and also improve the encoding of strong negative signal amplitudes, the whole population preferentially spikes during these amplitudes and therefore is locked to the underlying modulated signal.

To further increase the signal transmission quality the cells can be uncoupled by a source of noise, here slow noise (τ_*n*_ = 100 ms) is used which has previously been shown to be beneficial to signal transmission (Gerstner, 2000). In contrast to the case with large firing-rate (Figure 4), the slow noise had the opposite influence on the transfer functions, slightly increasing gain and phase for higher frequencies especially in the GrC model populations (Figures 5B1,B2). Noise furthermore enhanced the fidelity over the whole frequency range and especially counteracted the VAF drop of the GrC population at higher frequencies thus increasing the quality of input signal reconstruction (Figure 5B4) for standard and push-pull coding.

The mean population carrier-rate *F*_0_ of the granule cell model, estimated during activity without modulatory input, was 1.4 spikes/s without noise, and increased to 4.5 spikes/s with slow noise.

### Synaptic stimulation

Synaptic stimulation allowed us to examine the effects of signal transmission and combination through potentially non-linear synaptic processes and their interaction with intrinsic cellular properties.

#### Information transmission by individual types of synapses

The previous analysis probes only the effects of neuron intrinsic dynamics. We now investigate the effects of synaptic properties on information transmission for spiking inputs. For this all model cells have been connected with detailed models of AMPA, NMDA, and GABA_A_ synapses (Solinas et al., 2010) possessing dynamic synaptic properties such as presynaptic facilitation/depression and postsynaptic depression.

As described in Materials and Methods a low-passed white noise input signal was transformed into an input spike train with an input carrier-rate of 40 spikes/s using an ideal integrate-and-fire (iIF) neuron (τ = ∞) and the default modulation amplitude was *a* = 1. To separate the effects of input signal coding scheme and synaptic processing on the overall response of the complete GrC model, the transfer functions and fidelity of the generated input spike trains and of the resulting AMPA and NMDA conductance signals were analyzed separately (Figure 6).

Figures 6A1,A2 shows that, as expected, the transfer function for a single spike train and a population of *N*_*in*_ = 40 input spike trains showed a flat gain and phase. *N*_*in*_ = 40 is equivalent to the total input to a population of *N* = 10 model cells with 4 synapses each. Similar to before (Figure 3C3), the fidelity (Figure 6A3) of a single iIF (blue line) is degraded at high frequencies due to the large modulation amplitude of *a* = 1 but is flat for the population response (green solid line).

In contrast, the gain when the excitatory synapses were included (Figures 6B1,C1) featured strong dynamics, especially for AMPA, with a dip at ~2 Hz and resonance at ~10 Hz that can be accounted for by the complex synaptic dynamics. Especially the dip can be explained by presynaptic facilitation/depression as it disappeared when these are removed from the simulations (data not shown). Furthermore, due to their slower dynamics, NMDA synapses had a higher gain drop and phase delay (~−4 dB and −30° at 20 Hz) (Figures 6C1,C2) compared to AMPA synapses (~−1.5 dB and −20° at 20 Hz) (Figures 6B1,B2). In contrast to AMPA and NMDA, GABA synapses showed a constant gain decrease (Figure 6D1) but phase properties (Figure 6D2) similar to NMDA synapses. However, the VAF for all three synapses (Figures 6B3,C3,D3) showed no apparent difference to the VAF of the iIF coded spike input train indicating no significant information loss at the synaptic stage. This suggest that all these dynamic features as found in the transfer function are in fact linear since they can be counteracted by the linear Wiener filter and thus do not affect the reconstruction. However, this is only true if the modulation amplitude is not too large, as investigated further below.

#### Combined neuronal and synaptic effects on signal transfer

Next, the overall response of the GrC model with both excitatory and inhibitory inputs (see Materials and Methods) was analyzed. As described in Materials and Methods the excitatory synapses received trains with the same modulation signals but different mean input carrier-rates with mean *F*_0*in*_ of 40 spikes/s. The modulation amplitude was set to *a* = 1 to obtain the best signal to noise amplitude ratio, the initial size of the population was *N* = 10 (Figures 7A1–A3).

Panels A1,A2 show that the gain and phase of the transfer function for GrC with AMPA and NMDA excitation only exhibit the strong dynamics as seen in AMPA and NMDA transfer functions (Figures 6B,C1,C2) and the VAF is reasonably high with a mean of 95.2% for *N* = 10 (A3). When the synaptic conductance is decreased from its original value in the model (50%) (green lines), resulting in *F*_*eff*_ = ~40 spikes/s, the overall gain is shifted to lower values, the phase lag increases and VAF is decreased at higher frequencies due to the reduced firing-rate.

From here on all parameters are chosen to produce a mean effective firing-rate of *F*_*eff*_ = ~40 spikes/s.

Adding inhibition in the form of unmodulated constant spike trains with a rate of *F*_*I*_ = 6 spikes/s to all inhibitory synapses leads to an overall gain shift to lower values but also to an increased resonance at ~10 Hz and to a phase advance. More importantly however the VAF shows large dips at multiples of the rate of inhibition at 6 Hz. The reason for these VAF drops can be easily observed in the spike pattering as seen in single cell voltage traces (Figure 7B). This effect severely impairs the signal transmission quality at multiples of 6 Hz since the pattering is now part of the carrier signal, which concurs with the input signal modulation at these frequencies. Note that this influence of constant inhibition on signal transmission properties could not be inferred by looking at gain and phase alone (Figures 7A1,A2).

There seem to be two natural ways in which this severe deterioration in performance due to aliasing with the inhibitory input spike trains could be addressed by the biological system. Firstly natural signals would have intrinsic variability, which we can model by using spike trains with a higher CV, e.g., Poisson coded inhibitory spike trains instead of iIF trains (Figure 7A3, dotted orange lines). Secondly there might be variation in rate between individual inhibitory inputs, which we model by choosing *F*_*I*_ to be normally distributed with, e.g., 2 · *std*(*F*_*I*_) = 0.5 · *m*(*F*_*I*_) (solid red line).

In all cases inhibition, especially with Poisson input (dotted lines), has a similar effect on the VAF to that seen previously for slow noise (Figure 4): the VAF is lower for low frequencies. This indicates that, due to the slow synaptic properties of the GABA_A_ receptors inhibition essentially acts as a source of slow noise in this case.

To keep VAF uniformly above 90% the population size had to be increased to *N* = 100 (Figure 7A4) (red line), which however does not have a strong improvement on the prominent VAF dip for constant inhibition (orange line).

#### Information combination in the granule cell

In previous simulations the same signal has been conveyed by excitatory synapses only. We thus continued our analysis by testing signal combination at excitatory synapses (Figure 8A), at excitatory and inhibitory synapses (Figure 8B) and signal transmission through inhibitory synapses alone (Figure 8C). In the following the rate of inhibition was normally distributed, all spike trains were created by iIF coding, *N* = 100, *a* = 1 and all other parameters are chosen to always produce a mean effective firing-rate of *F*_*eff*_ = ~40 spikes/s.

When GrC cells receive two different modulatory input signals (*x*_*A*_(*t*), *x*_*B*_(*t*)) on two excitatory synapses each, or on one and three synapses, as depicted by the illustration above column A in Figure 8, the respective combined signal of *x*(*t*) = 2*x*_*A*_(*t*) + 2*x*_*B*_(*t*) (dark red line) or *x*(*t*) = 1*x*_*A*_(*t*) + 3*x*_*B*_(*t*) (light red line) can be reconstructed from the population response with high fidelity [*m*(*VAF*) = 95 or 96%]. These results are only slightly lower than for the control case without signal combination (Figure 7A3, red line) [*m*(*VAF*) = 96.9%] and can thus be considered linear.

When injecting a modulatory signal not only through excitatory but also inhibitory synapses (see illustration above column B), the contribution of the inhibition is not known a priori. We assumed that the factor w of inhibitory contribution is given by the reconstructed signal *x*(*t*) = *x*_*A*_(*t*) − *w* · *x*_*B*_(*t*) with the highest mean VAF. Simulations were done for two configurations resulting in baseline condition of *F*_*eff*_ = ~40 spikes/s: (1) normal excitatory conductance and *m*(*F*_*I*_) = 6 spikes/s (Figure 8B, solid orange line) and (2) reduced (45%) excitatory conductance but increased rate of inhibition [*m*(*F*_*I*_) = 40 spikes/s] (dotted orange line). In both cases 1 and 2 the optimal inhibitory contribution *w* was found to be *w* = 0.23 which indicates a lower contribution from inhibitory signals than from excitatory signals. This low contribution can be mainly attributed to the low synaptic time constant of the inhibitory synapse (using a single-exponential inhibitory synapse with τ = 10 ms increases the contribution to *w* = 0.8, data not shown).

While for the combination of excitatory and inhibitory signals the fidelity [*m*(*VAF*) = 96.1%] comes close to the control [*m*(*VAF*) = 96.9%] if the inhibitory input frequency is increased (case 2). However, the fidelity for signal transmission through the inhibitory synapses alone is always lower (Figure 8C, blue lines) further suggesting that these synapses are less suited to transmit information.

#### Relation between maximum input frequency and firing-rate

While previous simulations have been conducted obeying to the requirements of the Nyquist sampling theorem (*m*(*F*_*eff*_) = ~40 spikes/s and input frequency cutoff = 20 Hz], we continued to explore the regime below this relationship using a population of *N* = 100.

In the following simulations we used two different effective firing-rate/cutoff configurations of *F*_*eff*_ = ~20 spikes/s with cutoff = 30 Hz and *F*_*eff*_ = ~3.5 spikes/s with cutoff = 5 Hz. For IF models and simulations with increased amplitude (*a* = 10) a constant inhibition *I*_*I*_ was added (see Materials and Methods) to achieve similar effective firing-rates.

As a benchmark, we first analyzed the exclusive effect of spike sampling on the transmission properties by simulating a population of ideal integrate-and-fire neurons (iIF) for *F*_0*in*_ = 20 spikes/s and cutoff = 30 Hz (Figure 9A). While, as before (Figures 6A1,A2), the transfer gain and phase are flat for all cases (not shown), the VAF (A1) starts to deteriorate at the Nyquist frequency of 10 Hz for a modulation amplitude of *a* = 1 (green line). Interestingly the application of the push-pull coding scheme does not improve the VAF for this case (dark orange line) suggesting that signal rectification is negligible, However, increasing the modulation amplitude to *a* = 10 leads to an expected rectification of negative signals causing decreased VAF and impaired reconstruction (A1,A2, light green lines). This however can be counteracted by deploying push-pull coding (A1,A2, light orange lines) which results in a highly increased VAF over the whole frequency range and improved reconstruction.

While applying push-pull coding to the iIF population does not improve the VAF for *a* = 1 it does improve signal transmission in the GrC population (Figure 9B, red and blue lines). On the contrary, increasing the modulation amplitude to high values (*a* = 10, light red lines) is not beneficial even with push-pull coding (*a* = 10, light blue lines) in contrast to the iIF model. The reason for this discrepancy can easily be seen when comparing the binned firing rate of GrC and iIF populations (Figure 9D). For iIF, increasing the amplitude from *a* = 1 (green line) to *a* = 10 (light green line) leads to a relative increase of the spike rate for positive signals and a rectification for negative signals. For the GrC population however, the resulting signal with *a* = 1 (red line) shows a high amplitude when the change of input signal is large (D, ^*^) (i.e., high frequency) due to the synaptic properties of facilitation. However, if the amplitude of the input signal is too large (*a* = 10, light red line) the consecutive synaptic depression leads to a drop during a sustained signal (i.e., low frequency) (D, ^**^). This consecutively causes an increased gain at high frequencies and the strong dampening of low frequencies provokes a potential erroneous reconstruction at low frequencies (B1, light red line) that cannot be counteracted even when push-pull coding is employed (B1, light blue line).

The behavior in the GrC population can be almost exclusively attributed to the synaptic properties: replacing the GrC cells by passive IF models (see Materials and Methods) shows that the qualitative behavior of the VAF is maintained (B, dotted lines).

In the second configuration we analyzed the signal transmission at lowered effective firing-rate of ~3.5 spikes/s with cutoff = 5 Hz for GrC and IF models (Figure 9C). Just as in the case before, GrC and IF model simulations are qualitatively similar without push-pull coding [*m*(*VAF*) 84.9% vs. 84.2% for GrC and IF, respectively] (solid and dotted brown lines) while push-pull coding (solid and dotted purple lines) the results for IF are slightly better [*m*(*VAF*) GrC 90.9% vs. IF 93.6 for GrC and IF, respectively].

To further test the effect of spiking inhibition, simulations have been repeated in the configuration *F*_*eff*_ = ~20 spikes/s vs. cutoff = 30 Hz and *F*_*eff*_ = ~3.5 spikes/s vs. cutoff = 5 Hz without spiking inhibitory input (Figure 9E). The firing-rate was solely adjusted by the constant inhibition *I*_*I*_ and relative variance *v* was increased to *v* = 0.7 and 1.4, respectively to achieve comparable firing-rate distributions. For high firing-rate simulation (*F*_*eff*_ = ~20 spikes/s) the VAF for GrC and IF models (dotted and solid red lines) are similar to the case with spiking inhibition (Figure 9, compare dotted and solid red lines in E and B1). This relationship however changes when the firing-rate is low (*F*_*eff*_ = ~3.5 spikes/s). While the fidelity of the IF model is comparable to the case before (Figure 9, compare dotted brown lines in E and C1), the GrC model simulation shows a strong drop in low frequency VAF with constant inhibition only (Figure 9, compare solid brown lines in E and C1). This suggest that as in the case before with current stimulation and slow noise (Figure 5) spiking inhibition through GABA_A_ synapses imposes a slow noise component which helps to counteract the intrinsic non-linear properties of granule cells at low frequencies.

During synaptic stimulation the measured carrier-rate *F*_0_ was always slightly larger than the effective firing-rate *F*_*eff*_ with, e.g., *F*_0_ = 24.7 vs. *F*_*eff*_ = 21.8 spikes/s (B1,B2, GrC, cutoff = 30 Hz, red lines) or 5.8 vs. 3.6 spikes/s (GrC, cutoff = 5 Hz, C1,C2, brown lines). Without inhibitory input however, *F*_0_ was lower than *F*_*eff*_ with *F*_0_ = 17.3 vs. *F*_*eff*_ = 20.1 spikes/s (E, GrC, cutoff = 20 Hz, red line) or identical with *F*_0_ = *F*_*eff*_ = 3.3 spikes/s (E, GrC, cutoff = 5 Hz, brown line).

To extend our analysis to different firing-rates we further estimated the relation of maximum white noise cutoff frequency that can be transmitted under different mean effective firing-rates *F*_*eff*_ while maintaining *m*(*VAF*) > 90% for several model cells, conditions and a population of *N* = 100 (Figure 10). For the case of the ideal integrate-and-fire neuron (A), the *F*_*eff*_ vs. cutoff closely follows a straight 1:2 relation for an amplitude of *a* = 1 with (orange line) and without push-pull coding (green line). For an increased amplitude of *a* = 10 this relation is even better than 1:6 (light orange line).

For the following *F*_*eff*_ vs. cutoff relations, adjusting the rate of spiking inhibition controlled the firing-rate. For the granule cell model (solid lines) the relations at low *F*_*eff*_ are slightly better than Nyquist frequency (2:1) or 1:2 when applying push-pull coding. However, these relations soon improve with increasing firing-rate and reach up to 1:6 and 1:2 with and without push pull coding, respectively. Interestingly the relations saturate with a maximal transmittable cutoff frequency at about 60 Hz. Repeating these simulations with the passive IF model (dotted lines) shows a close agreement at low firing-rates reaching the same maximum effectiveness of 1:6 and 1:2 but show saturation at a higher cutoff frequency of 90 Hz. This suggests that at low firing-rates the main contribution to the transmission properties comes from the synaptic properties and intrinsic properties only come into play for high firing rates of the GrC model.

The best signal transmission properties, in terms of cutoff vs. *F*_*eff*_ relation, for synaptically activated GrC and IF models in the open-loop case seem to be at cutoff = 40 Hz which relates to *F*_*eff*_ = ~20 spikes/s for one and *F*_*eff*_ = ~6 spikes/s for push-pull coding. Furthermore, for both GrC model and passive IF model the advantage of employing push-pull coding saturates at a firing-rate of 40 spikes/s, which is the mossy fiber input frequency.

## Discussion

This study has tested the signal transmission properties of model granule cells (GrCs) under simulated current and synaptic stimulation. Using direct spike transfer-function estimation in addition to a variance-accounted-for (VAF) measure we have been able to analyse signal transmission fidelity based on intrinsic membrane and synaptic properties. The main finding is that the detailed GrC model with realistic mossy-fiber synaptic inputs is capable of transmitting information faithfully and linearly in the frequency range of the VOR despite the existence of non-linear intrinsic and synaptic mechanisms (Arleo et al., 2010; Gandolfi et al., 2013).

Faithful signal transmission can be achieved simply if the model neurons are driven to fire at a rate at least twice the highest required frequency of modulation (here assumed to be 20 Hz), but lower firing-rates can also be adequate if a population of neurons is utilized especially in combination with push-pull coding. The exact number of neurons required for faithful transmission depends on the precise values of firing rate and noise. The model neurons are also able to combine excitatory and inhibitory signals linearly. Finally, in this “open-loop” case with no input-related Golgi-cell feedback, i.e., only uncorrelated feedback, the detailed GrC model can be replaced by a simpler (modified) integrate-and-fire neuron especially in the case of a high tonic firing rate.

We consider first the issues raised by our methods of analysis, then the implications of the findings for how floccular GrCs could code vestibular information in principle. Next we discuss the experimental evidence relevant to this coding in practice and finally consider the implications of the present findings for future experimental and modeling studies.

### Methods of analysis

Analysing information transmission by systems that use pulse (i.e., spike) frequency modulation is difficult (Bayly, 1968; Dayan and Abbott, 2001). Here we employ two methods, first a sampling-rate filter method for a direct estimate of the transfer function (Bendat and Piersol, 2010), and second an ideal observer Wiener filter method (Gabbiani and Koch, 1998) for estimating the fidelity of signal transmission using a variance-accounted-for (VAF) measure. This second method is important because a linear transfer function cannot represent the non-linear (and potentially noisy) spike generation process perfectly accurately, but it requires knowledge of the statistics of the input signals (Materials and Methods).

The first point we would make with this study is to show the benefits of combining direct estimation of the transfer function and variance-accounted-for statistics. First of all, by calculating the transfer function directly from the spiking output without any binning or fitting we avoid any assumptions about subsequent filtering characteristics. This on the other hand separates the act of signal reconstruction quality completely from the transfer function estimation: while commonly used procedures of fitting single-sinusoids to the spike rate of single neurons (see Materials and Methods, Figure 1A) or to the binned population response (Richardson et al., 2003) would equate to some form of signal quality estimation embedded in the transfer function estimation itself, this is not the case when using a direct estimate. To this end we introduced variance-accounted-for statistics that add valuable knowledge about signal transmission quality, e.g., loss of transmission quality due to artifacts induced by inhibition processes (Figure 7).

Since this form of analysis makes use of Wiener filters that are non-causal, the results first and foremost represent an analysis of the information that can be linearly recovered from the spike trains and represents an upper bound. However, since the Wiener filters (Figures 2A3,B3) only have finite support in the time domain, causality could also be achieved in neural systems by introducing a delay in the reconstruction process (Bialek et al., 1991).

### Coding of vestibular information by floccular granule cells—theoretical possibilities

#### Current stimulation

Comparing the transfer function to the fidelity suggests that the intrinsic ion-channels of GrC models resulting in gain resonance for sinusoidal fit (Figure 3A) or gain increase for the direct estimate (Figure 3B) do not present substantial non-linearities and in fact can be reversed by the Wiener filter procedure if the firing rate is high (40 spikes/s).

Furthermore, for high firing rates, the GrC model has been shown to behave similarly to an integrate-and-fire neuron in terms of transfer function and fidelity. The typical resonance at ~10 Hz (D'Angelo et al., 2001) as also seen in single sinusoid spike transfer function gain measurements (Figure 3A) can be easily reproduced by a simple spike-dependent current and does not have any influence on the fidelity (Figure 3C). With the direct estimate of the transfer function used in this study, the resonance is concealed by the bigger carrier-rate resonance but can be uncovered either by additive uncorrelated noise to each neuron (Figure 4) or by a spread of population carrier-rates that both reduce the carrier-rate resonance. A reduction of the carrier-rate resonance indicates a reduced “locking” of the population to the carrier-rate (Knight, 1972) and can have beneficial effects on the fidelity as shown below.

In the presence of noise, high modulation amplitudes are clearly required to produce a large signal-to-noise ratio. Since the firing rate cannot be driven below zero, very large modulation amplitudes however can lead to rectification effects. The addition of noise in turn reduces the “locking” therefore uncoupling population firing which minimizes the rectification. Both adverse factors of noise and high amplitude rectification thus mutually cancel each other to some extent (Figure 4C1).

In contrast to the high-firing-rate case, low-firing-rate simulations (Figure 5) gain and fidelity of the detailed GrC model is substantially lower than for the IF models indicating that the intrinsic ionic currents have a non-linear influence that rises with decreasing firing-rate. This suggests that due to the increased inter-spike interval, especially the input frequencies with shorter wave-length have time to interact with the dynamic sub-threshold properties (Richardson et al., 2003) leading to a decreased fidelity at high frequencies.

Especially in the low-firing-rate simulation the modulation is prone to rectification effects since the modulation amplitude is higher than the carrier-rate. While as before, additive noise helps to counteract rectification we also found that an additional mechanism termed push-pull coding where a second population receives the inverted signal is very beneficial for the fidelity. The biological evidence relating to this possibility is considered below.

#### Synaptic stimulation

Estimating the transfer functions of the synaptic processes alone suggest that the dynamic receptor kinetics (Nieus et al., 2006; Mapelli et al., 2009), do not lead to any non-linearities that could decrease signal transmission in the synapses themselves if the modulation amplitudes are within a reasonable range (*a* = 1 see below). This means that albeit the dynamics, the synapses act almost completely as linear filters that can be reversed by the Wiener filter procedure.

The fidelity of synaptically activated small granule cell populations is high with excitation alone and signal combination at excitatory synapses is approximately linear. There has been some discussion on the literature (e.g., Dean et al., 2010) as to whether activity at multiple synapses is required to drive granule cell output (the integrator hypothesis, e.g., Jörntell and Ekerot, 2006) or whether the cell behaves as a “detonator” in which activity at a single synapse is sufficient (Rancz et al., 2007). The granule cell model we investigate here has been tuned to act as an integrator at the single spike level (requiring simultaneous activation on at least three of its four inputs to produce an output spike, as indicated by data from D'Angelo et al. (1995). Despite this our results show that it responds faithfully and linearly to independent modulations in firing rate at individual synapses. Hence it seems that the integrator-detonator distinction is not relevant in the modulated firing rate regime discussed here (although it may become relevant in the burst coding regime).

The addition of GABAergic inhibition can lead to two phenomena. First of all, if the inhibition frequency is identical for all synapses, aliasing with the inhibitory input spike trains, due to the small numbers of synapses, leads to substantial degradation of the VAF at the rate of inhibition. These results indicate that synchronized neuronal inhibition compromises the signal transmission quality of MFR and thus is an argument against GoC synchrony. The easiest way a biological system could avoid this behavior is by incorporating variability in the spiking inhibition. Our results indicate that variation in tonic rate with low CV is more effective than high CV inhibititory inputs, e.g., Poisson.

The second phenomenon we observed is that, due to the slow synaptic properties of the inhibitory input it can act as a slow noise source in the case of open-loop simulations, which has potential positive benefits (see below).

We also found linearity for signals presented at inhibitory synapses. However, although inhibition is effective in controlling the tonic spike rate of granule cells, we found that the contribution of these synapse to arithmetic operations is only 1/4th of that of an excitatory synapse due to their slow synaptic dynamics. Thus, GABAergic synapses are less suited to transmitting information.

For a population of ideal IF neurons a very large modulation amplitude and push-pull coding dramatically increases the fidelity. For synaptically stimulated cells (GrC and IF) however, very large modulation amplitudes lead to a decrease in fidelity that can be explained by the strong adaptation in the synaptic AMPA and NMDA models (Figure 9B3). This suggests that the main modulation amplitude cannot be increased indefinitely to improve signal to noise ratio when synapses possess short-term plasticity.

The use of push-pull coding has its main beneficial effect on the fidelity if rectification of negative signals occurs (Figures 9A1,A2, *a* = 10, light yellow vs. light green lines). On the other hand, synaptic stimulation of GrC or IF models with a modulation amplitude of only *a* = 1 and thus no rectification in the input benefits strongly from push-pull coding (Figures 9,B1,B2,C1,C2, 10B) suggesting that rectification emerges due to the characteristic “down-sampling” of high mossy fiber inputs of 40 spikes/s to lower granular cell frequencies (see below). This is further confirmed by the observation that the main beneficial effect of push-pull coding vanishes when the input frequency is equal to the GrC or IF output firing-rate of 40 spikes/s (Figure 10B). This suggests that signal transmission at the granular layer stage would massively benefit from push-pull coding.

Overall, apart from two exceptions, the influence of the synaptic properties seems to predominate the signal transmission properties of granule cells in the open-loop case. The first exception can be found in the cutoff vs. firing-rate relation of GrC and IF models. Here the saturation, i.e., the point at which an increase in firing-rate does not improve the possible cutoff frequency, is lower for GrC than for IF models.

The second exception can be found during synaptic stimulation resulting in a low-firing-rate (Figure 9E). Here, in absence of spiking inhibition, intrinsic properties of granule cells come into account and have a negative influence on the fidelity. The inhibitory input effectively acts as a noise source that has beneficial effects on the fidelity comparable to the direct slow noise current during subthreshold tonic current stimulation (Figure 5). In the absence of noise, the large amplitudes of the modulatory input signal can lead to an effective firing-rate *F*_*eff*_ that is higher than the carrier-rate *F*_0_. This however also results in a decrease of fidelity due to the preferred encoding of large signal components (Figure 5A4). The addition of noise to the population decouples the spike-precision from the large signal components and thus increases the fidelity as especially seen for granule cells. In return, this decoupling leads to a carrier-rate *F*_0_ that is larger than the effective firing-rate *F*_*eff*_.

### Coding of vestibular information by floccular GRCs—experimental evidence

#### Mossy fiber signals to flocculus

Recordings from putative mossy fibers in the floccular complex (Voogd and Barmack, 2006) of awake primates have established the basic properties of their vestibular coding (Lisberger and Fuchs, 1978; Miles et al., 1980). First, the majority (70%) of floccular mossy fibers carry signals related to eye position and eye movement, with no detectable vestibular input. Of the remaining 30% that do carry vestibular signals about 50–75% also respond to eye velocity or position, so that overall vestibular only (VO) mossy fibers constitute 8–15% of the total. Secondly, VO mossy fibers have high tonic firing rates (average 40–50 spikes/s) and code sinusoidal head-velocity by modulation of their firing rate. Their average sensitivity to head velocity is 0.76 spikes.s^−1^/deg.s^−1^, so they would on average be driven to 100% modulation by peak head velocities of ~60 deg.s^−1^. For human subjects peak head velocities are ~35 deg/s for walking and ~75 deg/s for running (Grossman et al., 1988; Pozzo et al., 1990). Thirdly, approximately half the fibers increase firing when the head moved ipsilateral to the recording site (type I), and half for contralateral movement (type II).

Similar results have been obtained by recording from the cells of origin of floccular mossy fibers, which are located mainly in the vestibular nuclei (Zhang et al., 1993; Cheron et al., 1996; Voogd and Barmack, 2006) for both awake primate (Zhang et al., 1993) and cat (Cheron et al., 1996), and also by recording EPSCs from floccular granule cells in mice anesthetized with ketamine-xylazine (Arenz et al., 2008). For anesthetized mice, as for awake primates, head velocity was encoded by modulation of tonic EPSC frequency, with about half the recordings showing an increase with ipsilateral head movement, and half with contralateral. The response to changes in head velocity was generally linear, with no obvious short-term synaptic dynamics, although because the tonic EPSC frequencies were low (13 Hz, range 0 to ~36) they could easily be driven to zero in the non-preferred direction so introducing a non-linearity. Bayesian stimulus reconstruction was use to estimate that about 100 synapses would accurately encode the head-velocity signal. It was concluded that head “velocity information is represented linearly via bidirectional modulation of EPSC frequency and charge round a tonically active vestibular input” (Arenz et al., 2008, p. 979). It is likely that the low tonic frequencies observed in this study resulted from the anaesthetic, since recordings of VO neurons in the vestibular nuclei of awake mice give average resting rates of 45–58 spikes/s (Beraneck and Cullen, 2007; Medrea and Cullen, 2013).

Because all the above studies focussed on low-frequency (<1 Hz) modulation of head velocity, measurements of the high-frequency signal carried by floccular mossy fibers are not available. However, recordings from VO neurons in the macaque vestibular nucleus indicate that they respond well to modulation of head velocity up to 16 Hz, the highest frequency tested (Massot et al., 2011). It seems plausible that at least some of these neurons do project to the flocculus (Zhang et al., 1993; Cheron et al., 1996; Massot et al., 2011).

In summary the evidence concerning mossy-fiber input to the flocculus indicate that the assumptions made in the present study are reasonable, and would provide a basis for linear coding over a wide range of frequencies and head velocities [though it should be noted that rhesus monkeys display negligible velocity dependent non-linearities in VOR gain and phase up to 300 deg/s over a wide frequency range (5–26 Hz) (Huterer and Cullen, 2002)]. Moreover the evidence that about half the mossy fibers carrying vestibular signals are in phase with contralateral head velocity and half out of phase would be consistent with a push-pull coding scheme of the kind investigated here.

Finally, floccular mossy-fibers also influence GrCs indirectly via unipolar brush cells (UBCs). These have not been included in the present model, and the implications of their presence for future work are considered below.

#### Granule cell firing in the flocculus

At present only preliminary reports of floccular GrC firing patterns are available. In awake rabbits GrC are usually silent or have very irregular tonic firing rates, and show a wide variety of responses to Gaussian-profile changes in head-velocity that may signal “various aspects of head velocity and acceleration, eye position, saccades and timing” (Hensbroek et al., 2006). These responses often included high frequency bursts (up to ~700 spikes/s for 10–25 ms), which appear sometimes to be related to the direction of movement only, rather than its velocity or acceleration (Van Beugen et al., 2013). Similar patterns of response have been described for rabbits anesthetized with ketamine-xylazine (Hensbroek et al., 2005; Van Dorp et al., 2009), though here responses related to head acceleration can be seen more readily, in contrast to the responses of floccular mossy fibers that are primarily related to head velocity (Hensbroek et al., 2012).

These initial results suggest that in closed loop conditions, tonic inhibition from Golgi cells is typically high enough to offset the high tonic firing rates of mossy fiber inputs. If so, the analysis of push-pull coding above becomes particularly relevant. It may also be the case that for some GrCs the phasic inhibitory input from Golgi cell input enables of a form of differentiation, converting velocity into acceleration signals. This possibility is considered further below.

#### Granule cell firing in other areas

While there is no specific information on mean spike rates and whether cells in the flocculus show a background activity (i.e., activity without stimulation) that could function as a carrier-rate, there is concrete information in other areas. While background activity was found absent in C3 zone cells sensitive to cutaneous stimulation, cells sensitive to joint movement show a mean background activity of 6 spikes/s in the decerebrate, non-anesthetized cat (Jörntell and Ekerot, 2006). Furthermore, background activity was found in half of the granule cells sensitive to limb stimulation in lobules Crus Ic/II a/b of anesthetized rats with mean activity of 3.9 spikes/s (Holtzman et al., 2011) and mean effective spike activity in the uvula-nodulus during vestibular stimulation is 3.3 spikes/s in anesthetized mice (Barmack and Yakhnitsa, 2008). This suggests that at least in some areas of the cerebellum the inhibition is low enough to allow for carrier-rate spike activity. However, also in this regime push-pull coding is likely to be beneficial for signal transmission if the mossy fiber activity is higher than granule cell activity.

### Implications for future work

As indicated in the Introduction, the analysis of information transmission by GrCs with uncorrelated inhibition (open-loop mode) is an initial step toward understanding the properties of the complete granular layer, as modeled by Solinas et al. (2010). Here we consider only future work on GrC processing in open-loop mode.

#### Role of unipolar brush cells in vestibular processing

The present model has assumed that floccular GrCs receive their vestibular signals as direct mossy-fibers from external sources, such as floccular projecting neurons in the secondary vestibular nuclei. However, a substantial proportion of this mossy-fiber input is processed further before it reaches the GrCs, because it is relayed via unipolar brush cells (UBCs) which are particularly numerous in the vestibulo-cerebellum (e.g., Mugnaini et al., 2011). Although the exact nature of this further processing is unclear, a variety of evidence suggests that UBC responses are more diverse than their mossy-fiber inputs (Simpson et al., 2005a,b; Hensbroek et al., 2006; Barmack and Yakhnitsa, 2008; Kennedy et al., 2014), probably because they can generate delayed and prolonged responses to brief stimulation (Locatelli et al., 2013; Kennedy et al., 2014; Van Dorp and De Zeeuw, 2014).

Diversity of GrC responses is a central feature for adaptive-filter models of the cerebellum (e.g., Dean et al., 2010), and could in principle be generated by diversity in mossy fiber, UBC, or Golgi cell inputs or GrC intrinsic properties (Dean et al., 2010, 2013; Gao et al., 2012; Houston et al., 2012; Geborek et al., 2013; Spanne and Jorntell, 2013; Kennedy et al., 2014). Including UBCs (Subramaniyam et al., 2014) in the present model would help clarify their contribution to diversity in GrC vestibular responses.

#### Burst coding by granule cells

Recent reviews (Arenz et al., 2009; D'Angelo and De Zeeuw, 2009; Chadderton et al., 2014) have argued that GrC coding of sensory signals takes at least two forms, one the linear modulation of firing rate MFR considered here, the second a brief burst response to sudden changes in sensory (often tactile) input in the absence of tonic firing. Applying the present model to burst coding could address important questions such as reproducing the very high frequencies observed experimentally for GrCs, the sensory parameters that GrCs burst could in principle encode, and the kind of information that could be lost in non-linear burst coding.

#### Experimental test

The open loop model used here predicts that if both tonic and phasic inhibition from Golgi cells were blocked, then the GrC response to sinusoidal vestibular stimulation would be modulation around a tonic firing rate. It might prove feasible to test this prediction by recording from GrCs in awake animals where granular layer inhibition has been blocked.

## General conclusion

We began by asking two questions. Firstly, are the properties of the granular layer compatible with the computational requirements of high-level cerebellar models, e.g., the adaptive filter model, as far as information transmission (rather than information recoding) is concerned, concentrating on the VOR as a test case. Furthermore, we focused only on uncorrelated inhibitory feedback under the assumption that information lost in open-loop mode cannot be regained whatever the properties of the full network.

Our answer to this question is positive: there are plausible mechanisms by which small populations of granule cells can faithfully represent an vestibular-like input signal, combine different classes of input signals linearly over excitatory inputs, and combine them linearly with a feedback signal from Golgi cells over inhibitory inputs. The next step is to use the methods established here to investigate information recoding in the closed-loop regime to verify that the granular layer inputs can supply the rich recodings needed for motor control problems. A clue as to the nature of these required recodings comes from engineering applications where linear adaptive filters based on “tapped delay lines” are used; producing accurate delayed versions of the input signal (Dean et al., 2010). Although this is biologically implausible, suggested alternatives include recoding by spectral timing filters, in which the input is processed by progressively broader filters whose peak recedes in time, by banks of frequency tuned filters, or by bases of exponential filters of increasing time constants (Fujita, 1982). In addition the recoding should include components implementing non-linear recoding of the input if it is to deal with non-linear control problems (Dean et al., 2010).

Our second objective was to explain the relatively complex intrinsic and synaptic dynamics of these cells in functional terms. In fact we found that these dynamics did not convey substantial (or any?) advantages in the MFR regime. The granule cells proved to be equivalent to a very simple resonant IF neuron and the synaptic properties seem if anything to reduce performance in this context. Hence it seems likely that granule cells specialization is related to the other aspects of function, possibly to the requirements of hybrid processing in the granular layer. In particular they may subserve the need for fast and reliable processing of information represented by short bursts of spikes (Gandolfi et al., 2013).

### Conflict of interest statement

The authors declare that the research was conducted in the absence of any commercial or financial relationships that could be construed as a potential conflict of interest.
